# A Nonlinear Constitutive Model for Remoulded Fine-Grained Materials Used under the Qinghai–Tibet Railway Line

**DOI:** 10.3390/ma15155119

**Published:** 2022-07-23

**Authors:** Liang Dong, Shuang Tian, Changrui Yao, Xiao Han, Ke Wang

**Affiliations:** 1 Railway Engineering Research Institute, China Academy of Railway Sciences Corporation Limited, Beijing 100081, China; dongl123@163.com; 2 School of Civil Engineering, Harbin Institute of Technology, Harbin 150090, China; ycr_hit@163.com (C.Y.); hanx_hit@163.com (X.H.); kwang_hit@163.com (K.W.); 3 State Key Laboratory for Geomechanics and Deep Underground Engineering, China University of Mining and Technology, Xuzhou 221008, China; 4 Chongqing Research Institute, Harbin Institute of Technology, Chongqing 401135, China

**Keywords:** constitutive model, frozen soil, mechanical behaviour, static triaxial test, Qinghai–Tibet Railway

## Abstract

Using undrained triaxial shear tests, this study investigates the mechanical properties of fine-grained materials (silty clay and sand) which are extensively used for China’s Qinghai–Tibet Railway (QTR) under different confining pressures (*σ*_3_) and freezing temperatures (*T*). The results show that a reduction in *T* causes an increase in the shear strength and elastic modulus of all the materials tested in the present study. In addition, the freezing of the silty clay has no significant effect on the type of soil behaviour (strain-hardening), whereas the freezing of the sand changes its strain-hardening behaviour to strain-softening. Supposing that the deviatoric stress–strain curves of the silty clay and sand can be divided into two segments due to a reverse bending point, it was assumed that the first segment follows a hyperbolic function. Meanwhile, the second segment is also a hyperbola, with the reverse bending point as the origin and the residual strength as the asymptote. Accordingly, a nonlinear relation constitutive model that considers *σ*_3_ and *T* is derived. All model parameters are identified. The reasonability of the new model was verified using the test results of the materials. A comparison of the predicted and test results shows that this model can well simulate the deviatoric stress–strain response in the failure process of the tested materials. In particular, it can reflect the residual deviatoric stress after the materials’ failure.

## 1. Introduction

The territory covered by frozen soil in China is the third largest in the world and includes 2.15 × 10^6^ km^2^ of permafrost areas [1]. Many key projects in China, such as railways, highways, and distance pipelines were built in these regions [2]. Among them, the Qinghai–Tibet Railway (QTR) is the highest and longest plateau railway in the world at present [3,4,5]. However, it has been reported that a large amount of frost damage has occurred since the QTR was built [6]. Therefore, it was essential for engineering design and maintenance to systematically investigate the mechanical properties of the frozen remoulded fine-grained materials.

In previous studies, the strength and deformation properties of frozen soil under static conditions considering different influencing factors have been researched. The influence of freezing temperatures (*T*) on the physical and mechanical characteristics of silty clay has been studied by many researchers [7,8,9,10,11,12,13]. It has been shown that *T* could significantly improve the physical and mechanical properties of soils due to the formation of a rigid ice–soil matrix [14]. Li et al. [15], Lat et al. [16], and Xu et al. [17] investigated the effects of the confining pressure (*σ*_3_), T, and moisture content on the static strength, stiffness, and damage behaviour of frozen soil by a series of triaxial static tests. However, the *σ*_3_ of previous work on frozen soil was relatively high, limited by the test equipment. Due to the low *σ*_3_ of the subgrade filling under the actual rail transit subgrade load in cold regions, the test results of a high *σ*_3_ fail to meet the engineering needs.

To master the working state of frozen soil in engineering structures, it is necessary to select the appropriate material constitutive model for analysis according to the actual situation [18]. Based on the experimental results, many constitutive models have been proposed to analytically and numerically study the strength and stress–strain relationship in frozen soil [19,20,21,22]. The Duncan–Chang constitutive model has clear concepts and is easy to understand [23,24]; so, it is widely used in hydraulic and geotechnical engineering, because it can better reflect the nonlinear behaviour of soil [25,26,27]. However, the Duncan–Chang model is not suitable for the evaluation of the frozen soil stress–strain curve, because the softening process of frozen soil cannot be well simulated.

In the following sections, the deformational characteristics of the silty clay and sand under different *σ*_3_ and *T* are investigated using static triaxial tests. A simplified nonlinear constitutive model that can capture strain-softening behaviour is developed to analyse the effects of *σ*_3_ and T. Next, the parameters involved in the model are evaluated, and insights and conclusions are drawn from the results.

## 2. Materials and Methods

### 2.1. Study Area and Soil Properties

**Figure 1:**
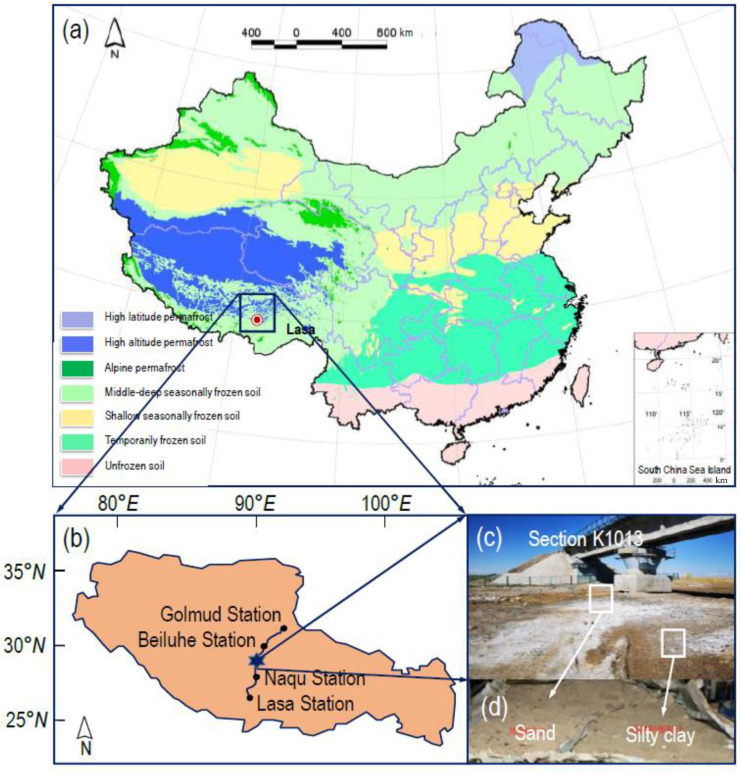
Remoulded fine-grained materials from Section K1013 along the Qinghai−Tibet railway: (**a**) frozen soil map of China; (**b**) Qinghai−Tibet railway; (**c**) section K1013 site; (**d**) remoulded fine-grained materials.

**Figure 2:**
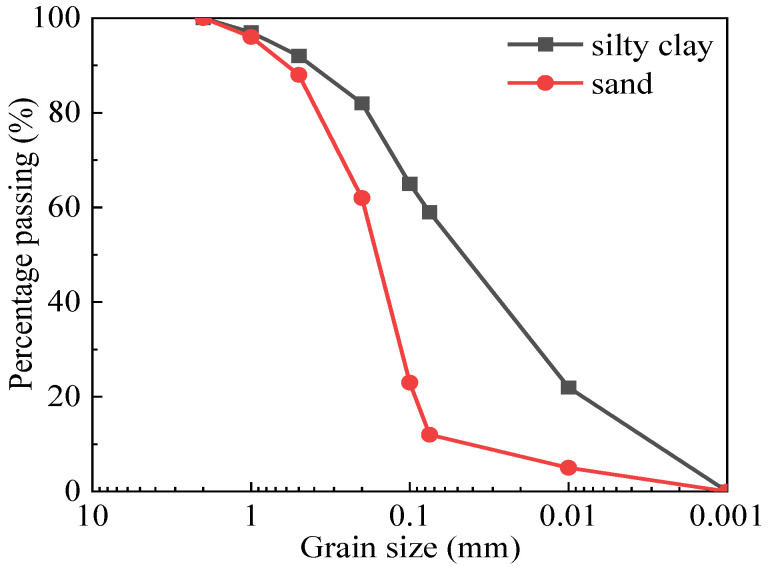
Particle size distribution curves of the remoulded fine-grained materials (silty clay and sand).

**Figure 3:**
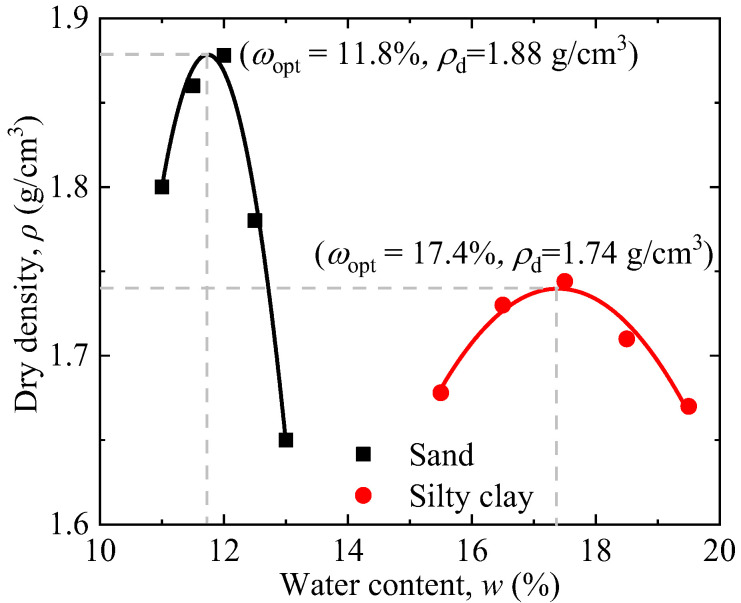
Compaction curve of the remoulded fine-grained materials (silty clay and sand) used in this study.

The silty clay and sand of the remoulded samples was collected within the range of 0.5 m under the shoulder at section K1013 along the Qinghai–Tibet Railway, the highest altitude railway in China’s permafrost regions (see Figure 1). The gradation curves of the soil sample are shown in Figure 2. Through laboratory tests, the optimal moisture content of the silty clay was found to be 17.4%, and the corresponding maximum dry density was 1.74 g/cm^3^ (see Figure 3). The optimal moisture content of the sand was 11.8%, and the corresponding maximum dry density was 1.88 g/cm^3^ (see Figure 3). The two fillings basically accorded with the typical characteristics of the density and optimal water content of silty clay and sand, respectively.

### 2.2. Sample Preparation

According to the Code for Soil Tests of Railway Engineering in China (TB10102-2010) [28], the method of sample compaction at different layers was adopted in the current study. The silty clay and sand obtained from the Qinghai–Tibet Railway was cleaned, dried, and sieved, and only the particles under a 2 mm diameter sieve were collected to make the remoulded samples. The samples were divided into five layers to compact, where the mass of each layer could be obtained considering 95% of maximum dry density (ρ_d, max_). Subsequently, purified water was added to reach the optimum moisture content (w). Later on, the prepared soil mixtures were kept in enclosed bags for 24 h to prevent evaporation. Lastly, the cylindrical material samples with a height of 200 mm and a diameter of 100 mm were compacted layer-by-layer using a standard proctor hammer. After compacting one layer, the layer interface was made sufficiently coarse to ensure the two layers were integrated. Then, the specimens were wrapped with rubber sleeves, and the top and bottom were covered with epoxy resin platen to prevent water evaporation.

### 2.3. Test Procedures

**Figure 4:**
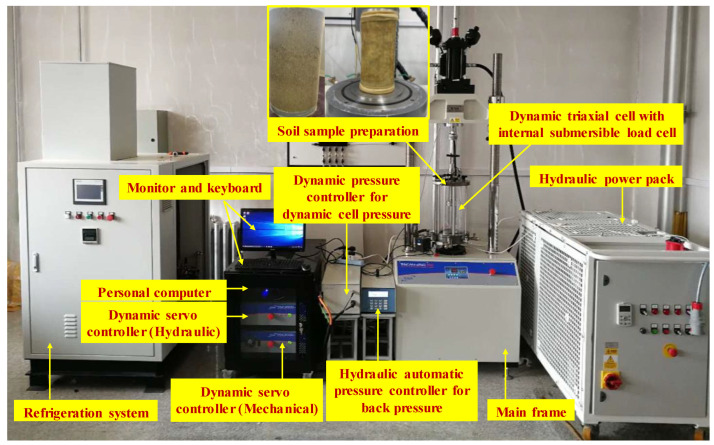
Cryogenic triaxial test system and the tested samples.

The dynamic triaxial tests were conducted on a fully automated Global Digital System (GDS), a cryogenic triaxial apparatus, illustrated in Figure 4. The system is a digitally controlled servo pneumatic system that controls two parameters: axial stress and confining pressure (*σ*_3_). The system incorporated a control and data acquisition system, which can maintain an auxiliary air receiver with a servo valve for cell pressure control, local deformation measurement in the vertical direction, and a submersible load cell measuring the applied axial load. The stable confining pressure ranged from 20 kPa to 4 MPa, and the maximum axial load and displacement were 40 kN and 85 mm, respectively.

According to Wang et al. [29], a closed system was adopted for the test program. A one-dimensional freezing–thawing model was employed to simulate the direction of freezing and thawing from the top to the bottom. To ensure one-dimensional freezing and thawing, only the sample top surface was exposed to the external environment. A 50 mm thick layer of insulating polystyrene was used to protect the perimeter and bottom of the cylinder.

The variation range in ground temperature along the Qinghai–Tibet Railway in China is roughly between −20 °C and 20 °C, with a large temperature difference. Therefore, the temperatures under the static loading condition were set to −10 °C, −5 °C, and −1 °C. The time needed to freeze the sample was determined by a pre-experiment test. A short resistance temperature detector probe was inserted at the centre of a pre-experiment test sample during the freezing process to show the variation in temperature. It revealed that 12 h was sufficient to freeze the sample to reach −10 °C with slight fluctuations, plus or minus 0.2 °C.

The test sample was isotropically consolidated at *σ*_3_ = 100 kPa, 150 kPa, or 200 kPa after the sample temperature was under the preset temperature for 12 h. After consolidation, the test specimen was then subjected to shearing at an axial strain rate (1.25 mm per min) until the strain of 15% was reached. The particular test scheme is shown in Table 1.

## 3. Experiment Results and Analysis

### 3.1. Stress–Strain Behaviour

**Figure 5:**
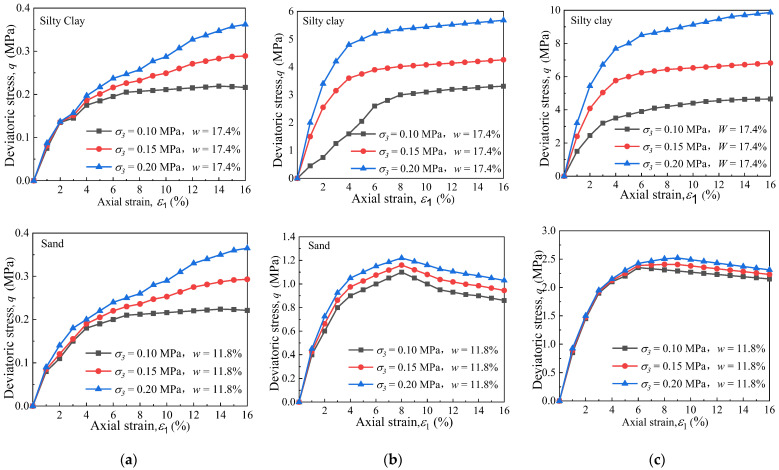
Dependence of deviatoric stress on the axial strain of tested remoulded fine-grained materials corresponding to different confining pressures: (**a**) *T* = −1 °C, (**b**) *T* = −5 °C, and (**c**) *T* = −10 °C.

To illustrate the mechanical behaviour of the remoulded fine-grained materials that were influenced by the freezing temperature and *σ*_3_, Figure 5 shows the typical stress–strain curves for the samples. The results show that the shear strength of the frozen silty clay was significantly greater than that of the frozen sand at the same conditions, especially at low temperatures. It is seen that all the stress–strain curves for silty clay were of the strain-hardening type, and the principal stress deviation had nonlinear growth with the increase in the axial strain, in which the shape of curves was hyperbolic. The stress–strain curves of the silty clay show that the shape of the stress–strain curve did not change with the variation in the temperature, but the degree of hardening was weakened. Decreasing the freezing temperature to −5 °C and −10 °C increased the shear strength of the silty clay by 1374% and 2258% with respect to the silty clay of −1 °C when the *σ*_3_ = 150 kPa.

As for the sand, when the freezing temperature was the same, the stress–strain curves’ variation tendency was similar with the different *σ*_3_. However, the stress–strain curves gradually changed to strain-softening as the freezing temperature decreased. Moreover, the reduction in the temperature from −1 °C to −10 °C caused an increase in the shear strength of 661% for the frozen sand under a *σ*_3_ of 150 kPa. It can be concluded that the influence of freezing on the increase in shear strength of the silty clay was much greater than that for sand.

### 3.2. Cohesion and Internal Friction Angle

**Figure 6:**
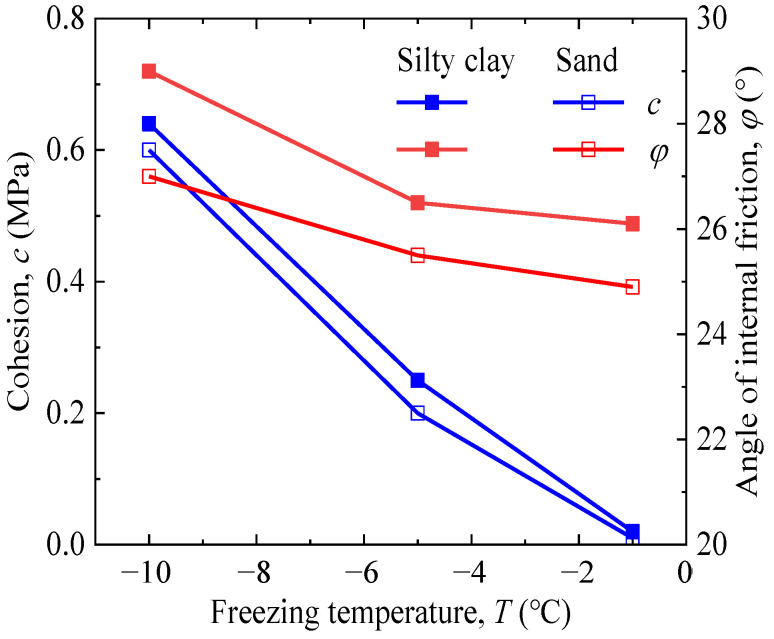
Variation in the cohesion and angle of internal friction versus freezing temperature.

The results in Figure 5 were processed using the Mohr–Coulomb strength criterion expressed by the “p-q” method to obtain the internal friction angle φ and cohesion c in Figure 6. According to Figure 6, the samples of silty clay with more water content than that of sand strengthened the bite friction during the freezing process; so, the cohesion and angle of the internal friction in silty clay were slightly greater than those in sand. Here, the cohesion increased by approximately 5900% and 3100% for silty clay and sand, respectively, comparing the samples at the temperatures of −1 °C and −10 °C. Similar to the cohesion, the reduction in temperature from −1 °C to −10 °C led to an 11.1% and 8.4% increase in the internal friction angle of silty clay and sand, respectively. It is noted that the rate of increase in the cohesion and angle of internal friction due to the reduction in temperature was almost identical for the silty clay and sand.

### 3.3. Elastic Modulus

**Figure 7:**
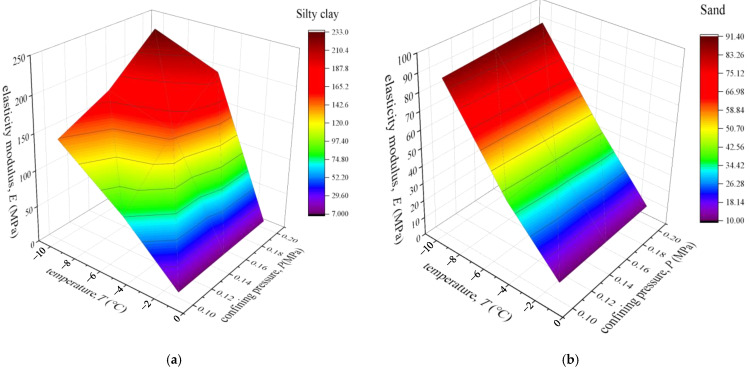
Variation in the elastic modulus versus freezing temperature and confining pressure: (**a**) silty clay and (**b**) sand.

The effect of *σ*_3_ and freezing temperature reduction on the modulus of elasticity (E_e_) of the silty clay is presented in Figure 7a. The values of E_e_ for the silty clay under the temperature of −1 °C for *σ*_3_ values of 100, 150, and 200 kPa were approximately 7.5, 9, and 10 MPa, respectively. After the freezing temperature decreased to −5 °C, the growth in E_e_ was approximately 900%, 1567%, and 1880%, respectively. As the freezing temperature continued to decrease to −10 °C, the values of E_e_ increased by 1793%, 1844%, and 2230% with respect to the silty clay at −1 °C for the three *σ*_3_ values, respectively.

Moreover, a decrease in freezing temperature led to a significant increase in the E_e_ of sand. Figure 7b presents the effect of the freezing temperature reduction on the E_e_ of the sand tested under *σ*_3_ values of 100, 150, and 200 kPa at the freezing temperature of −10 °C, −5 °C, and −1 °C. As expected, the E_e_ of sand generally increased with the decreasing freezing temperature. The growth in the E_e_ of sand under the freezing temperature of −10 °C relative to the sand at −1 °C was approximately 775%, 743%, and 715%, respectively, for the three *σ*_3_ values.

## 4. A Nonlinear Constitutive Model for Remoulded Fine-Grained Materials

### 4.1. Establishment of the Model

**Figure 8:**
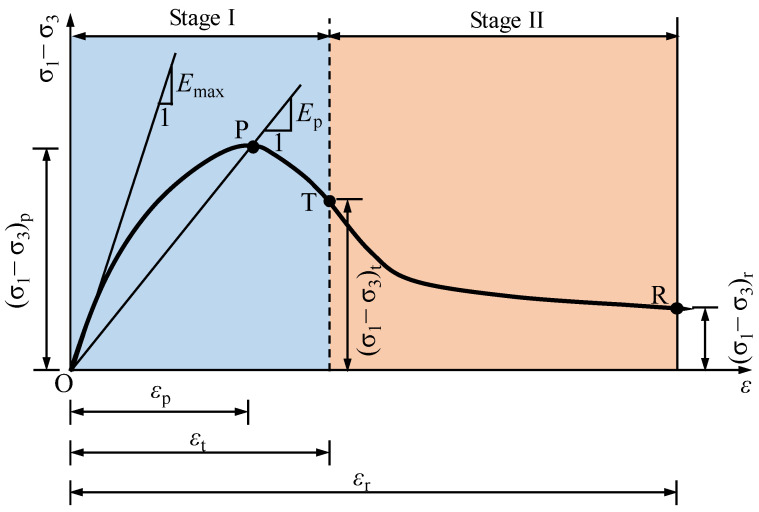
Schematic diagram of a nonlinear constitutive model for the remoulded fine-grained materials.

As shown in Figure 8, a softening-type stress–strain model with a high degree of accuracy can be used to approximate the nonlinear stress–strain curves for the remoulded fine-grained materials under different freezing temperatures [30]. After careful inspection, the piecewise continuous functions for the proposed constitutive model are expressed as follows:

(1a) $$R_{\sigma }=D_{\beta }\frac{1+b_{2}R_{\varepsilon }}{1+b_{3}R_{\varepsilon }}\cdot \frac{R_{\varepsilon }}{1+b_{1}R_{\varepsilon }}\left(R_{\varepsilon }\leq R_{\varepsilon t}\right)\;\;\;\;$$

(1b) $$R_{\sigma }=R_{\sigma t}-\frac{R_{\varepsilon }-R_{\varepsilon t}}{b_{4}+b_{5}\left(R_{\varepsilon }-R_{\varepsilon t}\right)}\;\;\;\;\;\;\;\;\;\;\;\;\;\;\;\;\;\;\;\;\;\;\left(R_{\varepsilon }>R_{\varepsilon t}\right)\;\;$$

where *D_β_*, *b*_1_, *b*_2_, *b*_3_, *b*_4_, and *b*_5_ are undetermined coefficients that can be obtained from the experimental results. The parameters *R_σ_*, *R_ε_*, *R_σt_*, and *R_εt_* are defined by Equation (2a) through (2d) as follows:

(2a) $$R_{\sigma }=\frac{\sigma_{1}-\sigma_{3}}{{\left(\sigma_{1}-\sigma_{3}\right)}_{p}}$$

(2b) $$R_{\varepsilon }=\frac{\varepsilon }{\varepsilon_{p}}$$

(2c) $$R_{\sigma t}=\frac{{\left(\sigma_{1}-\sigma_{3}\right)}_{t}}{{\left(\sigma_{1}-\sigma_{3}\right)}_{p}}$$

(2d) $$R_{\varepsilon t}=\frac{\varepsilon_{t}}{\varepsilon_{p}}$$

where (*σ*_1_ − *σ*_3_) is the deviator stress; *σ*_1_ and *σ*_3_ are the major principal stress and minor principal stress, respectively; *ε* is the axial strain; (*σ*_1_ − *σ*_3_)*_p_* and *ε_p_* are the deviator stress and deviator strain, respectively, at the peak point (i.e., point P in Figure 8); and (*σ*_1_ − *σ*_3_)*_t_* and *ε_p_* are the deviator stress and the deviator strain, respectively, at the inflection point (i.e., point *T* in Figure 8).

(1) Coefficient determination for Equation (1a).

To accurately represent the relationship between (*σ*_1_ − *σ*_3_) and *R_ε_*, the terms on both sides of Equation (1a) were multiplied by (*σ*_1_ − *σ*_3_)_p_ and Equation (1a) was rewritten as follows:

(3) $$\sigma_{1}-\sigma_{3}=D_{\beta }{\left(\sigma_{1}-\sigma_{3}\right)}_{p}\frac{1+b_{2}R_{\varepsilon }}{1+b_{3}R_{\varepsilon }}\frac{R_{\varepsilon }}{1+b_{1}R_{\varepsilon }}$$

The compressive strengths of the fillers are assumed to satisfy the Mohr–Coulomb failure criterion. The relationship between the compressive strength (*σ*_1_ − *σ*_3_)*_p_* and the confining pressure *σ*_3_ is described by the following equation:

(4) $${\left(\sigma_{1}-\sigma_{3}\right)}_{p}=\frac{2\left(c_{p}\cos\varphi_{p}+\sigma_{3}\sin\varphi_{p}\right)}{1-\sin\varphi_{p}}$$

in which *c_p_* and *φ_p_* are the Mohr–Coulomb strength parameters.

Next, Equation (4) is substituted for (*σ*_1_ − *σ*_3_)*_p_* in Equation (3) to yield

(5) $$\sigma_{1}-\sigma_{3}=D_{\beta }\frac{2\left(C_{p}\cos\varphi_{p}+\sigma_{3}\sin\varphi_{p}\right)}{1-\sin\varphi_{p}}\frac{1+b_{2}R_{\varepsilon }}{1+b_{3}R_{\varepsilon }}\frac{R_{\varepsilon }}{1+b_{1}R_{\varepsilon }}$$

where *D_β_* = *D* × *β*, with *β* as a modified coefficient and *D* = *E*_max_/*E_p_*. *E*_max_ and *E_p_* (i.e., *E_p_* = (*σ*_1_ − *σ*_3_)*_p_*/*ε_p_*) are the initial tangent modulus at the original point (i.e., point O in Figure 8) and the secant modulus at the peak point, respectively. The determination of *E*_max_ is equivalent to the determination of *E*_max_ in the Duncan–Chang model [23].

Performing the differentiation on Equation (1a) produces the following expression

(6) $$\frac{dR_{\sigma }}{dR_{\varepsilon }}=D_{\beta }\frac{1}{\left(1+b_{1}R_{\varepsilon }\right)\left(1+b_{3}R_{\varepsilon }\right)}\left[\frac{\left(b_{1}-b_{2}\right)R_{\varepsilon }}{1+b_{3}R_{\varepsilon }}+\frac{1+b_{2}R_{\varepsilon }}{1+b_{1}R_{\varepsilon }}\right]$$

In addition, *R_σ_* can be considered as a function of the parameter *R_ε_* in Equation (1a) and attains its extreme value at the peak. Thus,

(7) $$\frac{dR_{\sigma }}{dR_{\varepsilon }}=0$$

Combined with Equations (6) and (7),

(8a) $$\frac{\left(b_{1}-b_{2}\right)}{1+b_{3}}+\frac{1+b_{2}}{1+b_{1}}=0$$

(8b) $$b_{1}=\frac{b_{2}b_{3}+2b_{2}+1}{b_{3}-b_{2}}$$

In this case, *R_σ_* = 1 and *R_ε_* = 1 at the peak point based on their definitions in Equation (2). Thus, these values are substituted into Equation (1a) to obtain

(9) $$1=D_{\beta }\cdot \frac{1+b_{2}}{1+b_{3}}\cdot \frac{1}{1+b_{1}}$$

According to Equation (1a), *R_εt_* and *R_σt_* may be expressed at the inflection point as follows:

(10) $$R_{\sigma t}=D_{\beta }\frac{1+b_{2}R_{\varepsilon t}}{1+b_{3}R_{\varepsilon t}}\cdot \frac{1}{1+b_{1}R_{\varepsilon t}}$$

By combining Equations (9) and (10), *b*_2_ and *b*_3_ can be expressed as

(11a) $$b_{3}=\frac{R_{\varepsilon t}-1+\beta_{t}\left(1+b_{1}R_{\varepsilon t}\right)-\beta_{p}\left(1+b_{1}\right)R_{\varepsilon t}}{\left[\beta_{p}\left(1+b_{1}\right)R_{\varepsilon t}-\beta_{t}\left(1+b_{1}R_{\varepsilon t}\right)\right]R_{\varepsilon t}}$$

(11b) $$b_{2}=\beta_{p}\left(1+b_{1}\right)\left(1+b_{3}\right)-1$$

where $\beta_{t}=\frac{R_{\sigma t}}{D_{\beta }R_{\varepsilon t}}$ and $\beta_{p}=\frac{R_{\sigma p}}{D_{\beta }R_{\varepsilon p}}$.

Three undetermined coefficients (i.e., *b*_1_, *b*_2_, and *b*_3_) are included in Equation (11). When the original *b*_1_ is given, *b*_2_ and *b*_3_ are evaluated using Equations (11a) and (11b). Subsequently, *b*_2_ and *b*_3_ are substituted into Equation (8) to obtain a new value for *b*_1_. This procedure can be repeated by using the original *b*_1_ until the given and recalculated *b*_1_ are identical.

(2) Coefficients of determination for Equation (1b).

Similarly, the terms on both sides of Equation (1b) are simultaneously multiplied by (*σ*_1_ − *σ*_3_)*_p_*. Next, Equation (1b) is combined with Equation (2a) to (2d) as follows:

(12) $$\sigma_{1}-\sigma_{3}={\left(\sigma_{1}-\sigma_{3}\right)}_{t}-\frac{\varepsilon -\varepsilon_{t}}{\frac{1}{E_{p}}b_{4}+\frac{1}{E_{p}\varepsilon_{p}}b_{5}\left(\varepsilon -\varepsilon_{t}\right)}$$

If we let

*b*_4_ = *E_p_A* (13a)

*b*_5_ = *E_p_ε_p_B* (13b)

then, Equation (12) is rewritten as

(14) $$\sigma_{1}-\sigma_{3}={\left(\sigma_{1}-\sigma_{3}\right)}_{t}-\frac{\varepsilon -\varepsilon_{t}}{A+B\left(\varepsilon -\varepsilon_{t}\right)}$$

The tangent modulus (*E*) at any point in the first segment of the curve (i.e., *R_ε_* ≤ *R_εt_*) can be directly represented as

(15) $$E=\frac{d\left(\sigma_{1}-\sigma_{3}\right)}{d\varepsilon }=\frac{d\left(\sigma_{1}-\sigma_{3}\right)}{dR_{\sigma }}\cdot \frac{dR_{\sigma }}{dR_{\varepsilon }}\cdot \frac{dR_{\varepsilon }}{d\varepsilon }$$

The expressions of *R_σ_* and *R_ε_* can be rewritten as

(16a) $$\frac{d\left(\sigma_{1}-\sigma_{3}\right)}{dR_{\sigma }}={\left(\sigma_{1}-\sigma_{3}\right)}_{p}$$

(16b) $$\frac{dR_{\varepsilon }}{d\varepsilon }=\frac{1}{\varepsilon_{p}}$$

Equation (16) is substituted into Equation (15) to obtain

(17) $$\frac{d\left(\sigma_{1}-\sigma_{3}\right)}{d\varepsilon }=\frac{{\left(\sigma_{1}-\sigma_{3}\right)}_{p}}{\varepsilon_{\sigma }}\cdot \frac{dR_{\sigma }}{dR_{\varepsilon }}=E_{p}\frac{dR_{\sigma }}{dR_{\varepsilon }}$$

Equation (6) is substituted into Equation (17), and the tangent modulus in the first segment of the curve can be expressed as

(18) $$\frac{d\left(\sigma_{1}-\sigma_{3}\right)}{d\varepsilon }=D_{\beta }\frac{E_{p}}{\left(1+b_{1}R_{\varepsilon }\right)\left(1+b_{3}R_{\varepsilon }\right)}\left[\frac{\left(b_{1}-b_{2}\right)R_{\varepsilon }}{1+b_{3}R_{\varepsilon }}+\frac{1+b_{2}R_{\varepsilon }}{1+b_{1}R_{\varepsilon }}\right]$$

In addition, *D_β_* = *D* × *β*, *D* = *E*_max_/*E_p_*, Equation (18) can be rewritten as

(19) $$\frac{d\left(\sigma_{1}-\sigma_{3}\right)}{d\varepsilon }=\frac{E_{\max}}{\left(1+b_{1}R_{\varepsilon }\right)\left(1+b_{3}R_{\varepsilon }\right)}\left[\frac{\left(b_{1}-b_{2}\right)R_{\varepsilon }}{1+b_{3}R_{\varepsilon }}+\frac{1+b_{2}R_{\varepsilon }}{1+b_{1}R_{\varepsilon }}\right]$$

Therefore, the tangent modulus (${\bar{E}}_{t,1}$) at the inflection point in the first segment of the curve can be obtained from Equation (19).

(20) $${\bar{E}}_{t,1}=\frac{E_{\max}}{\left(1+b_{1}R_{\varepsilon t}\right)\left(1+b_{3}R_{\varepsilon t}\right)}\left[\frac{\left(b_{1}-b_{2}\right)R_{\varepsilon t}}{1+b_{3}R_{\varepsilon t}}+\frac{1+b_{2}R_{\varepsilon t}}{1+b_{1}R_{\varepsilon t}}\right]$$

In addition, the tangent modulus (${\bar{E}}_{t,2}$) at the inflection point in the second segment of the curve can be expressed from Equation (12) by using Equation (21) in terms of the definition of the derivative.

(21) $${\bar{E}}_{t,2}=\underset{\varepsilon \to \varepsilon_{t}}{\lim}\frac{\left(\sigma_{1}-\sigma_{3}\right)-{\left(\sigma_{1}-\sigma_{3}\right)}_{t}}{\varepsilon -\varepsilon_{t}}=\underset{\varepsilon \to \varepsilon_{t}}{\lim}\frac{-1}{A+B\left(\varepsilon -\varepsilon_{t}\right)}=-\frac{1}{A}$$

To ensure a continuous condition at the inflection point, the tangent modulus at the inflection point in the two segments of the curve should be equivalent, which indicates that ${\bar{E}}_{t,1}={\bar{E}}_{t,2}$ at the inflection point. Therefore, Equation (21) is substituted into Equation (20) to obtain

(22) $$A=-\frac{1}{\frac{-E_{\max}}{\left(1+b_{1}R_{\varepsilon t}\right)\left(1+b_{3}R_{\varepsilon t}\right)}\left[\frac{\left(b_{1}-b_{2}\right)R_{\varepsilon t}}{1+b_{3}R_{\varepsilon t}}+\frac{1+b_{2}R_{\varepsilon t}}{1+b_{1}R_{\varepsilon t}}\right]}$$

Consequently, *b*_4_ can be evaluated when the value of *A* is substituted into Equation (13a).

When the deviatoric stress (*σ*_1_ − *σ*_3_)*_r_*, which is the residual strength and its corresponding strain *ε_r_* at any point in the second segment of the curve, is substituted into Equation (2), *R_σr_* and *R_εr_* can be evaluated, and *b*_5_ can be obtained using Equation (23). In this study, the residual deviatoric stress (*σ*_1_ − *σ*_3_)*_r_* is selected when the strain *ε_r_* is equivalent to 20%.

(23) $$b_{5}=-\frac{b_{4}+\frac{R_{\varepsilon r}-R_{\varepsilon t}}{R_{\sigma r}-R_{\sigma t}}}{R_{\varepsilon r}-R_{\varepsilon t}}$$

### 4.2. Determination of Model Parameters

Six parameters need to be determined from Equation (1) in the proposed model, including the parameters related to the basic elastic properties (*D_β_*), the peak stress state (*b*_1_, *b*_2_, and *b*_3_), and the residual stress state (*b*_4_ and *b*_5_). Most of these model parameters can be conveniently obtained through the monotonic triaxial tests incorporating *σ*_3_ and *T*. The initial small stress–strain data can be adopted to calculate the *D_β_*. The residual stress state parameters (*b*_4_ and *b*_5_) can be evaluated through the monotonic triaxial tests results at the residual stress state using Equations (13) and (23). The peak state parameters (*b*_1_, *b*_2_, and *b*_3_) can be determined by a trial-and-error process using Equations (8) and (11), i.e., by comparing the model predictions and laboratory data. Furthermore, the values of the abovementioned undetermined coefficients with the corresponding values are shown in Table 1.

### 4.3. Model Verification

**Figure 9:**
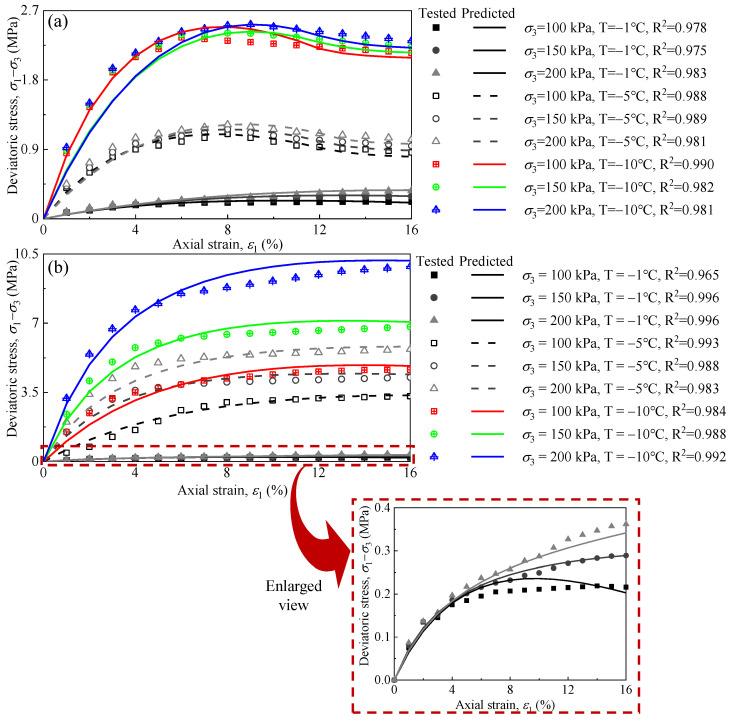
Comparison of the predicted and tested data of the tested remoulded fine-grained materials: (**a**) sand and (**b**) silty clay.

The test results of the sand and silty clay subjected to different *σ*_3_ and *T* are used to validate the proposed model. Figure 9 shows the predicted stress–strain of the sand and silty clay compared to the laboratory observations. Note that the model predictions match well with the test data, and the strain-hardening and strain-softening of the samples subjected to different *σ*_3_ and *T* have been captured very well.

## 5. Discussion

### 5.1. Sensitivity Analysis of the Parameters

**Figure 10:**
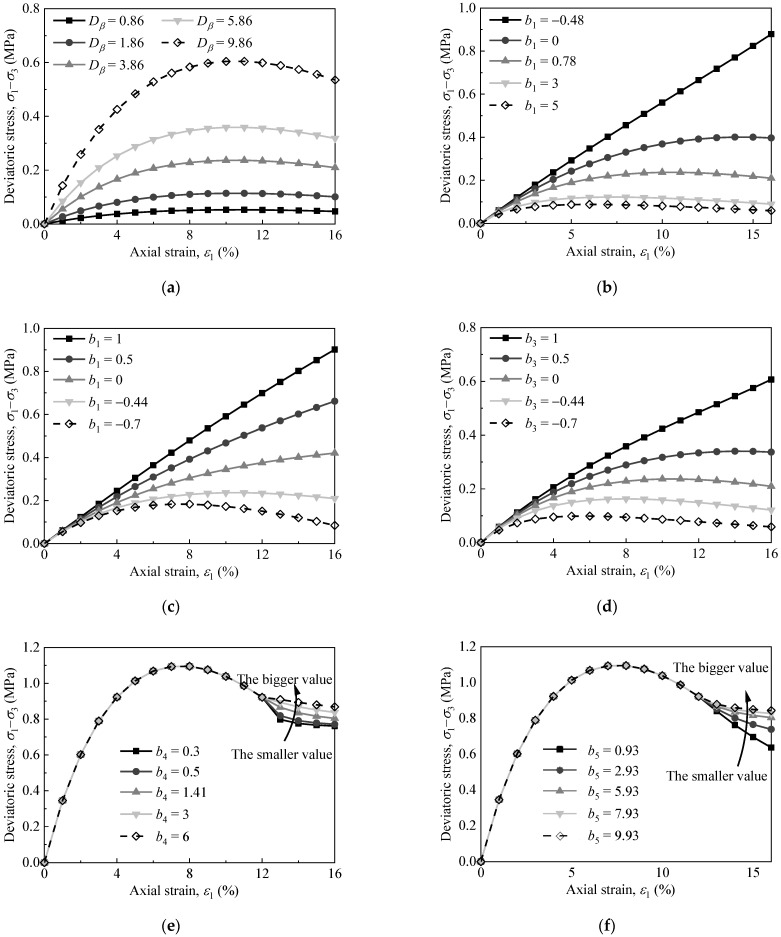
Deviatoric stress–strain curves of the proposed model for different values of parameters: (**a**) *D_β_*, (**b**) *b*_1_, (**c**) *b*_2_, (**d**) *b*_3_, (**e**) *b*_4_, and (**f**) *b*_5_.

In Equation (1a), let *b*_1_ = 0.78, *b*_2_ = −0.44, and *b*_3_ = 0.21; thus, Figure 10a demonstrates the deviatoric stress–strain curves of the proposed constitutive model for different values of the parameter *D_β_*. It can be seen from Figure 10a that the change in the value *D_β_* has no obvious influence on the shape of the deviatoric stress–strain curve. However, with the increase in the value *D_β_*, the initial elastic modulus of the model increases gradually, indicating that the parameter *D_β_* mainly reflects the initial elastic modulus of the materials; indeed, the larger the parameter *D_β_* is, the higher the initial elastic modulus will be.

Furthermore, in Equation (1a), let *D_β_* = 3.86, *b*_2_ = −0.44, and *b*_3_ = 0.21; let *D_β_* = 3.86, *b*_1_ = 0.78, and *b*_3_ = 0.21; and let *D_β_* = 3.86, *b*_1_ = 0.78, and *b*_2_ = −0.44; Thus, Figure 10b,d presents the deviatoric stress–strain curves of the proposed constitutive model for different values of the parameters *b*_1_, *b*_2_, and *b*_3_, respectively. Figure 10b,d show that the change in the values *b*_1_, *b*_2_, and *b*_3_ has a great influence on the shape of the deviatoric stress–strain curve. With a decrease in the values *b*_1_ and *b*_3_ and an increase in the value *b*_2_, the stress–strain curves gradually change from strain-softening to strain-hardening. This indicates that the parameters *b*_1_, *b*_2_, and *b*_3_ mainly reflect the peak strength and brittleness degree of the materials’ failure. The larger the parameters *b*_1_ and *b*_3_ are, and the smaller the parameter *b*_2_ is, the more obvious the brittleness failure characteristic of the materials will be.

In addition, it can be seen from Figure 10 that the variation in *b*_4_ and *b*_5_ only changes the shape of the residual stress state. With an increase in *b*_4_ and *b*_5_, the residual stress increases, indicating that the failure characteristic changes from brittleness to ductility.

### 5.2. Comparison with the Existing Model

**Figure 11:**
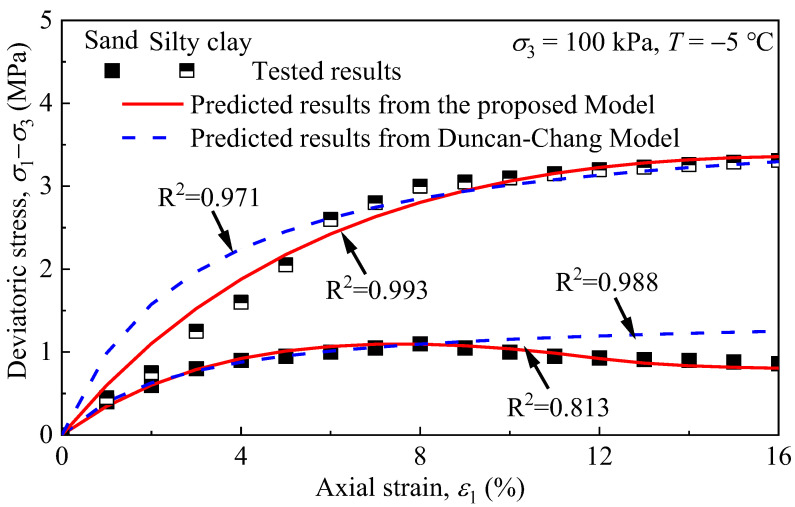
Comparison of the predicted and tested data of the tested remoulded fine-grained materials under *T* = −5 °C and *σ*_3_ = 100 kPa.

In order to further illustrate the rationality of the proposed model, the triaxial compression test results of silty clay and sand, with *σ*_3_ = 100 kPa and *T* = −5 °C, were employed to validate it. Figure 11 presents the comparison of the theoretical curves from the proposed model and the Duncan–Chang model. It can be seen from Figure 11 that the theoretical curves from the Duncan–Chang model had good agreement with the test data at the prepeak region but poor agreement at the postpeak region for the mechanical behaviours of the materials. In particular, it could not reflect the residual deviatoric stress after the materials’ failure, while the theoretical curves from the model proposed in the current study conform well with the test data in both the prepeak region and postpeak region, no matter what the shape of the stress–strain curves. Therefore, the proposed model has better adaptability.

## 6. Conclusions

This paper presented the results of a comprehensive experimental investigation to study the effect of confining pressure (*σ*_3_) and freezing temperature (*T*) on the mechanical behaviour of remoulded fine-grained materials (silty clay and sand) used under the Qinghai–Tibet Railway line. Accordingly, a nonlinear constitutive model, which could reflect the postpeak softening behaviour was established. Conventional monotonic triaxial tests incorporating *σ*_3_ and *T* carried out in the current study were used to evaluate all the model parameters. The salient outcomes of the model are summarized below.

(1) All the silty clay exhibited a strain-hardening type of stress–strain curve, but the sand under the temperatures of −5 °C and −10 °C showed strain-softening. Under the same test conditions, the shear strength of the silty clay was greater than that of the sand. In all cases, the cohesion (*c*) and angle of internal friction (*φ*) of the silty clay were greater than that of sand. Furthermore, the modulus of elasticity of the materials tested increased due to freezing and the temperature reduction.
(2) A practical constitutive model was developed to represent the nonlinear, stress-dependent, and inelastic stress–strain behaviours of the fillers subjected to freezing and thawing. This model incorporated three important aspects of the stress–strain behaviour, including nonlinearity, strain-dependency softening, and inelasticity. A simple technique was used to interpret the test results and conveniently determine the six parameters in the model.
(3) The triaxial test results of the remoulded fine-grained materials were employed to evaluate the reasonability of the proposed model established in this paper. A comparison of the predicted and test results showed that this model could well simulate the deviatoric stress–strain response in the failure process of the tested materials. In particular, it could reflect the residual deviatoric stress after materials’ failure.
(4) This study analysed the behaviour of the fillers with optimum water content that were exposed to the freeze–thaw cycles to develop a constitutive model. If appropriate experimental results are available, the parameter values in the proposed model can be derived from the triaxial test results. Therefore, additional experiments should be conducted to investigate other parameters, such as the temperature, duration of freezing and thawing, and the moisture content and compactness of the fillers, which are important characteristics of fillers in regions that are subjected to seasonal freezing and thawing.

## Figures and Tables

**Table 1:** Summary of test schemes and the corresponding model parameters.

Soil Type	Test No.	Confining Pressure (kPa)	Temperature (°C)	*b*_1_	*b*_2_	*b*_3_	*b*_4_	*b*_5_	*D*_β_	The Patterns of the Stress–Strain Curves
Sand	S1	100	−1	0.78	0.44	0.213	-	-	3.86	Strain-hardening
	S2	150		0.54	0.46	0.165	-	-	3.30	Strain-hardening
	S3	200		0.39	0.43	0.174	-	-	2.84	Strain-hardening
	S4	100	−5	0.78	0.38	0.0036	1.41	5.93	2.91	Strain-softening
	S5	150		0.54	0.25	0.0077	1.22	6.39	2.53	Strain-softening
	S6	200		0.39	0.33	0.0057	1.16	6.71	2.24	Strain-softening
	S7	100	−10	0.78	0.25	0.0142	3.65	11.95	2.41	Strain-softening
	S8	150		0.54	0.33	0.0192	2.60	9.43	2.35	Strain-softening
	S9	200		0.39	0.39	0.0446	1.83	8.44	2.40	Strain-softening
Silty clay	SC1	100	−1	1.26	0.42	0.19	-	-	4.67	Strain-hardening
	SC2	150		3.73	0.29	0.24	-	-	4.94	Strain-hardening
	SC3	200		4	0.02	0.16	-	-	4.28	Strain-hardening
	SC4	100	−5	1.26	0.29	0.17	-	-	3.74	Strain-hardening
	SC5	150		3.73	0.16	0.26	-	-	7.08	Strain-hardening
	SC6	200		4	0.14	0.12	-	-	6.54	Strain-hardening
	SC7	100	−10	1.26	0.29	0.47	-	-	4.65	Strain-hardening
	SC8	150		3.73	0.16	0.26	-	-	7.08	Strain-hardening
	SC9	200		4	0.17	0.14	-	-	6.82	Strain-hardening

## Data Availability

Not applicable.
